# “It's a fight to get anything you need” — Accessing care in the community from the perspectives of people with multimorbidity

**DOI:** 10.1111/hex.12571

**Published:** 2017-05-24

**Authors:** Julia W. Ho, Kerry Kuluski, Jennifer Im

**Affiliations:** ^1^ Institute of Health Policy Management and Evaluation University of Toronto Toronto Ontario Canada; ^2^ Collaboratory for Research and Innovation Sinai Health System Lunenfeld‐Tanenbaum Research Institute Toronto Ontario Canada

**Keywords:** access to care, chronic disease management, multimorbidity, multiple chronic disease, patient experience, qualitative methods

## Abstract

**Background:**

There is a growing interest in redesigning health‐care systems to better manage the increasing numbers of people with multimorbidity. Knowing how patients experience health‐care delivery and what they need from the health‐care system are critical pieces of evidence that can be used to guide health system reforms.

**Objective:**

The purpose of this study was to understand the challenges patients with multimorbidity face in accessing care in the community, and the implications for patients and their families.

**Methods:**

A secondary analysis of qualitative data was conducted on semi‐structured interviews with 116 patients who were receiving care in an urban rehabilitation facility in 2011. Exploratory interpretive analysis was used to identify themes about access to care.

**Results:**

Challenges occurred at two levels: at the health system level and at the individual (patient) level. Issues at the health system level fell into two broad categories: availability of services (failing to qualify, coping with wait times, struggling with scarcity and negotiating the location of care) and service delivery (unreliable care, unmet needs, incongruent care and inflexible care). Challenges at the patient level fell into the themes of logistics of accessing care and financial strain. Patients interacted and responded to these challenges by: managing the system, making personal sacrifices, substituting with informal care, and resigning to system constraints.

**Conclusion:**

Identifying the barriers patients encounter and the lengths they go to in order to access care highlights areas where policy initiatives can focus to develop appropriate and supportive services that are more person and family‐centred.

## 1 INTRODUCTION

Chronic conditions, such as cardiovascular diseases, cancer and chronic respiratory diseases, have reached epidemic proportions and constitute leading causes of death in the developed world.^1^ Given the prevalence and burden of chronic illness, chronic disease management is a priority of many health systems worldwide. In Canada, 74% of people over the age of 65 experience multimorbidity (two or more chronic conditions simultaneously).^2^ As our population ages rapidly, and life expectancy and survival rates improve, the effects of multimorbidity are likely to drive health‐care utilization needs and associated costs.^3^, ^4^, ^5^, ^6^ Multimorbidity has major implications for patients, their families and society. Therefore, redesigning models of care towards systems that have a chronic disease focus is necessary to help combat the challenges associated with managing multimorbidity.

The needs and health problems of patients with multimorbidity tend to carry a high treatment burden due to increased use of health services,^7^ complicated treatment and medication regimes,^8^ and interactions with care providers across an array of settings.^9^ Patients may experience frustration with care providers, as well as, lack confidence in the overall health system.^10^ At times, patients may fail to utilize services all together, even when available. However, there is limited understanding of why this occurs in the existing literature. More is to be learned about the various factors that influence the way in which people with multimorbidity access (or fail to access) the health‐care system when needed.

The concept of access is discussed widely in the health service performance and utilization literature^11^, ^12^ and is considered an important indicator of care quality.^13^, ^14^ Scholars have conceptualized “access” in a variety of ways. In the 1970s and 1980s, the early period of health services utilization research, researchers described access as the “fit” between patients and services.^15^ Penchansky and Thomas^15^ theorized that patient satisfaction was determined by whether services met patient expectations, and the level of satisfaction was an indicator of “fit” or degree of accessibility to services. Andersen^12^ distinguished the degree of service utilization as “realized access” rather than a measure of “potential access”. Donabedian's^16^ evaluation framework for health quality suggests the characteristics of health‐care resources as instrumental in determining access or service use by potential patients. A recent scoping review on the concept of access by Levesque et al.^17^ proposes a “patient‐centred” view of access, defined as “the opportunity to reach and obtain appropriate health care services in situations of perceived need for care” (p. 4). Levesque et al.^17^ framework considers the process of access as the interface of supply‐side factors of health care (approachability, acceptability, availability and accommodation, affordability, appropriateness) and demand‐side characteristics of populations (ability to perceive, ability to seek, ability to reach, ability to pay, ability to engage).

“Access” to health care and its associated issues (eg, equity, health outcomes, institutional structures) is a continuous area of interest for researchers and health‐care decision‐makers. Indeed, understanding the shortcomings of health systems in meeting patients' needs and more broadly the patient experience has become an important focus of health services administration and research. Health‐care providers and policy makers are increasingly concerned with the patient experience as this concept is seen as an indicator of quality in health‐care systems.^18^, ^19^ The emergent scholarship on patient experience, in the 1980s and 1990s, tended to assume proxy measurements, such as rates of patient satisfaction,^20^, ^21^ captured the necessary information regarding access to care. This approach is still routinely used today. Standardized surveys that measure patient satisfaction with settings of care,^22^ treatment modalities^23^ and interactions with care providers^24^ are ubiquitous administrative tools in quality improvement initiatives. While it may be useful to have a general sense of these factors, we argue these metrics do not give a full picture of the patient experience because they are largely defined by the “supply‐side” (the care provider‐side) perspective, while the “demand‐side” (patient‐side) conceptualization of what is meaningful about access is largely absent.

This article presents the view that patients have to “work” or actively engage and negotiate with health policies and services in order to manage multimorbidity. While we acknowledge that, in general, all patients may also engage in this “work”, we suggest that how patients with multimorbidity experience health services requires closer examination because they are an “experienced” subpopulation of patients due to their multiple encounters with the health system and their long‐term use of health services. As intense users with multiple needs, the challenges these patients face are likely to bring to the forefront salient issues relevant to improving the patient experience.

Patients with multimorbidity and their families often have to navigate a web of programmes in the community, many of which have their own entry criteria, waiting lists and assessment tools. Patients may have little choice but to negotiate these complexities all while managing health conditions that affect them and their caregivers mentally, emotionally and physically.^25^

This inquiry focuses on access in community services (home care, community services, primary care) which arguably are some of the most crucial settings in the chronic disease management journey. The purpose of our study was to both illuminate the types of challenges, as well as, gain insight into how patients deal with these challenges.

## 2 METHODS

This article describes a secondary analysis of 116 patient interviews drawn from a mixed methods cross‐sectional study conducted at a 404‐bed urban rehabilitation and complex continuing care facility in Toronto, Canada in 2011. The intent of the primary study was to understand the needs and experiences of patients with complex chronic disease, and to use this knowledge to inform quality improvement and policy making within the facility and at similar facilities. The patients were interviewed by experienced qualitative researchers who met with patients one‐on‐one, and asked a combination of open‐ and closed‐ended questions informed by a complex chronic disease framework comprised of five health dimensions (medical/physical health, mental health, demographics, social capital, health and social experiences), the result of a scoping literature review on multimorbidity and expert consultation conducted by the same research team.^26^ The patients in this study had an average of five health conditions and several illness symptoms including problems with daily activities, pain management and mental health issues. The qualitative results of the primary study revealed three broad themes that represent the important components of care delivery: components of the care plan, care capacity and quality and the patient–provider relationship.^27^

The senior author (KK) of this article was the lead investigator for the primary study. The lead author (JH) led an earlier study^28^ based on a secondary analysis of data from the primary study but did not participate in the original study. The third author (JI) was not involved in the primary study. The data were reviewed by the lead author for overall quality, appropriateness for the secondary research question and presence of thick descriptions.^29^ The data analysed were drawn from responses to the open‐ended question: “Please describe any challenges you have had with accessing health services in the past” and any references in other parts of the interview where the patient described challenges in access to health services. Asking about access issues in “the past” was appropriate given that the patients interviewed were currently in hospital. Although interviewing patients during a vulnerable time may elicit particular perspectives on health services, we acknowledge this context and suggest that understanding how patients view health‐care access during a time when they are engaged in the health system (while in hospital) captures patient reflections during an “embedded” state in their health‐care journey.

NVivo software version 10 was used to generate a node report that included all patient comments related to challenges in accessing care in the community from the entire pool of participants. We determined the themes inductively through a systematic process of dividing the data into meaning units, organizing the units into domains, constant comparison of meaning units to the final generation of themes that reflected the relational nature of the phenomena.^30^, ^31^ Themes were determined using exploratory interpretive analysis, a qualitative analysis approach that explores meanings and explanations of phenomena.^32^

The data were analysed by a three‐member research team. The lead author (JH) is a nurse who has worked in the home and community care sector for a number of years and has conducted previous qualitative research on patients with multimorbidity. The senior author of this article (KK) is an experienced qualitative researcher whose main programme of research is patient and caregiver experience in the health system. The third author (JI), is also a trained qualitative researcher and focuses her research on palliative care and ethics.

We were attentive and conscious throughout the analysis process of how our personal views and experiences may have influenced our interpretations of the data. We acknowledge that our clinical experiences, research projects and previous contacts with patients with multimorbidity guides us in the construction of meaning in the transcripts. The lead author and third author independently coded the transcripts, and then met with the senior author on multiple occasions to discuss the study's findings. The process of independent coding by two researchers, in addition to the prolonged engagement garnered by a core researcher from the original study is intended to promote credibility and fittingness of the findings.^33^ The themes went through several iterations where the team members had in‐depth discussions to ensure that particular themes were not over/under emphasized and that the themes were representative of all findings. A consensus was reached between the three researchers to determine the final themes presented in this article.

## 3 RESULTS

**Figure 1:**
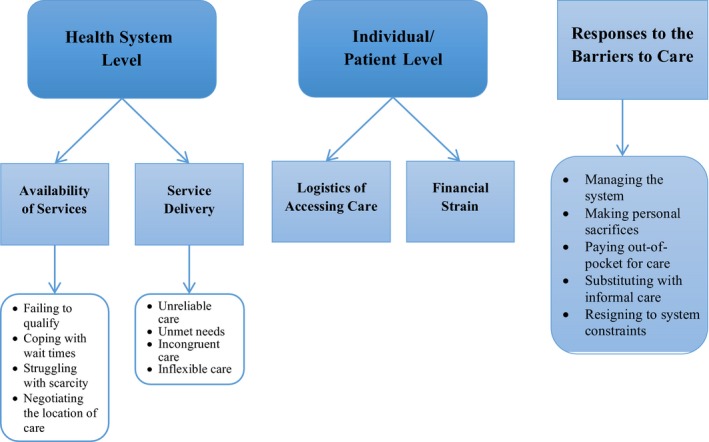
Themes of access to care in the community for patients with multimorbidity

We determined the following broad categories and associated themes to accessing health‐care services in the community (see figure 1). Challenges occurred at two levels: at the health system level and at the individual (patient) level. Issues at the health system level fell into two broad categories: availability of services (failing to qualify, coping with wait times, struggling with scarcity and negotiating the location of care) and service delivery (unreliable care, unmet needs, incongruent care and inflexible care). Challenges at the patient level were categorized as logistics of accessing care and financial strain. Patients interacted and responded to these challenges by: managing the system, making personal sacrifices, substituting with informal care and resigning to system constraints.

### 3.1 Health system level challenges

#### 3.1.1 Availability of services

Some patients identified systemic barriers (eg, programme policies and guidelines) to access that required a significant amount of effort to deal with. These patients characterized “access” as a process of attainment because although the types of services they required were available, due to programme rules, the services were *not* available to *them*. The results of this effort or work were captured in the themes: failing to qualify, coping with wait times, struggling with scarcity and negotiating the location of care.

##### Failing to qualify

Some patients described how they “failed” to meet the minimum threshold for service enrolment. A 51‐year‐old patient, recovering in hospital from knee surgery described her experience with accessing post‐discharge services:

*I'm asking for a month's assistance, I don't qualify. I know I don't qualify because of the age category that I'm in… I'm too young. I'm too young..That's disheartening*. *(EH 007)*

While some patients did not meet age requirements for programmes, others did not qualify due to their financial status:

*I tried to get into assisted living in <place> or <place> and I was declared that I had too much money and I didn't qualify. I made $20 000 more last year than their cut‐off. So as a result, I didn't get an apartment. …Hopefully in the near future, they will change their… Because just because you have a little more money doesn't mean you don't need help*. *(CMB 022)*

Being “just nicely over the dividing line” (CMB048), yet not quite meeting the threshold of eligibility, was a source of disappointment for patients. Furthermore, the work or the inability to prove themselves “worthy” of care was a common sentiment amongst these patients.

##### Coping with wait times

Some patients commented on long waits for health services. This patient described her reaction to the news that she had to wait months before diagnostic tests:

*…Like how do I cope with every day? And there was just no answer there. So the delay in the medical system, the long waits that you have to get a specialist to see you, the long waits that you have just to get the tests are frustrating*. *(CMB045)*

Patients also cited long queues and quotas for rehabilitation treatments, such as physiotherapy and speech language pathology. While waiting for health services, patients found they were ill‐equipped to manage their health conditions and experienced symptoms like pain and lack of mobility. These factors contributed to feelings of uncertainty in their future health.

##### Struggling with scarcity

Patients who were already receiving health services experienced cutbacks or reductions in the hours/visits they received from formal care providers. A 65‐year‐old patient described the changes in her Personal Support Worker (PSW) services:

*They get cut back. Like with the PSW, I was given 3 times a week and then when they start getting towards the end of the budget, the money's running down, then you get cut back. And sometimes you get it back, sometimes you don't…* *(CMB 025)*

Primary care was another area where patients experienced challenges accessing care. For example, some patients described difficulty in finding a family doctor who was taking on new patients. Also, some patients portrayed a sense of weariness in the struggle to deal with the limitations of the health system.

##### Negotiating the location of care

Some patients who lived in rural communities experienced challenges accessing health care. For example, a patient from a town in Northern Ontario (largely rural) commented: *“I wish that physio was not such a challenge… I'm looking at 45 minutes to an hour travel in some direction from where I live” (EH007)*. Other patients described the problems they had accessing community‐based care due to service boundary restrictions. One patient said she needed foot care from a senior's health centre but was told she did not live within their catchment area, so was not eligible for the care. Negotiating the administrative complexities associated with location of care, in addition to the geographical distribution of services, necessitates patients to not only acquire knowledge about how the health system works, but also, as illustrated by this theme, this interaction needs to be reconciled with the patients' health and social conditions.

In summary, the broad category of “availability of services” described here depicts how health policies affect the daily activities of managing health conditions for patients with multimorbidity and brings attention to the forms of engagement enacted by patients: “failing to qualify,” “coping with wait times,” “struggling with scarcity,” and “negotiating the location of care”.

### 3.2 Service delivery

The category of “service delivery” represents the challenges that some patients had with their formal care providers. The themes of “unreliable care,” “unmet needs,” “incongruent care,” and “inflexible care,” characterize the types of problems that patients encountered.

#### 3.2.1 Unreliable care

Patients were frustrated with service providers who were late for or missed appointments:

*But the home care worker didn't show up so I missed that appointment. And by that time I was having really bad pain in my legs. And I don't think I did much on the Saturday. Maybe I went out to get cigarettes but that was it. And then I fell Saturday night*. *(SH016)*

Patients relied on their care providers to follow through with home visits because daily routines, such as a spouse handing‐off care to a formal care provider, were contingent on timely care provision.

#### 3.2.2 Unmet needs

Some patients were unable to obtain appropriate levels of care in the community. As illustrated below, finding a long‐term care home that meets a patient's needs can be challenging:

*Yes. First of all, I can't afford semi‐private. And second, there's so many filled up and not enough nursing homes. The one down in <street>, in <street>, they were going to take me. And I was all ready to go and then they changed their mind… They said I'm too tall. And then the others, he's too heavy. I was waiting for one to come to in and say that, “Well, he eats too much,” but that hasn't happened yet. “Hey, we can't afford to feed him. We'll just throw him some hay in a barn and leave him there.”* *(CMB020)*

Some patients commented that the physical spaces of health‐care programmes were ill‐conceived for people who had special needs such as pain management issues and physical disabilities. One patient reported she attended a pain clinic where she had to endure three hours of education while sitting in a confined space. This experience prompted the patient to raise the question: “*how much they [the pain management program] really understand about pain?”* (CMB 020). Similarly, a wheelchair‐bound patient questioned the suitability of the accommodations in her living space for her functional ability:

*I mean I was asked if my kitchen was wheelchair accessible. I said yes, it's wheelchair accessible but it's not all accessible to me. But that part was ignored*. *(CMB047)*

#### 3.2.3 Incongruent care

Some patients disagreed with their care providers about issues that included: treatment goals, types of treatments and diagnoses. Comments that fell into this theme conveyed the sense that patients lacked confidence and trust in their care providers as the following comment illustrates, *“I don't have a lot of confidence with the doctor that I have right now. I wish I could change but there's nowhere to go”* (EB 003). Also, some patients cited communication‐related challenges with care providers that led one patient to suggest that “*she [doctor] doesn't want to be my doctor and she's trying to figure out how she can get out of being my doctor (*CMB026).*”* The misalignment between patients' expectations and care provision was a source of stress for patients and the sentiment that this relationship was like “fighting the system” was prevalent in this theme. Some patients also framed these problems as unresolvable or there were no alternatives to their current care provision.

#### 3.2.4 Inflexible care

Some patients discussed the bureaucratic nature of service delivery. For example, one patient questioned the process in which her care was delivered:

*But why do I have to pay for a nurse that comes for blood pressure and a nurse that comes the same day to do my blood? One nurse should do both*. *(CMB 001)*

Some patients described how frustrated they were with certain home care practices. For example, after patients received their prescribed allotment of physiotherapy treatments, patients' family members were expected to take over treatments, a practice which patients felt was unreasonable as they believed their family members did not have the capacity to take over these tasks.

Challenges in service delivery were experienced by patients as care that was “unreliable,” “unable to meet needs,” “incongruent with expectations,” and “inflexible”. In short, some patients believed they received suboptimal care from the health system.

### 3.3 Patient level challenges

Patient level challenges, or issues that pertained to individual circumstances rather than at a system level, included: “the logistics of accessing care” and “experiencing financial strain”.

#### 3.3.1 Logistics of accessing care

Arranging for and travelling to services was problematic for some patients. A patient who had physical disabilities could not travel to her prescribed pain clinic appointments due to transportation problems and physical access barriers:

*But you see, I didn't go back because of some of first barriers, like the cost of the parking. And then they said to me, “Well, can't you take like Go Train and subway?” …Well, yes but then I have to walk around the subway trying to find the elevators and the escalators because I can't do stairs. And then the whole thing was so mind boggling for me that I gave up. I gave up easily* *(CMB022)*

A patient who ambulated in a wheelchair described the difficulty travelling to and from appointments in the city:

*And if I go over anything more than 2 inches, it would take the bottom out. And there's no place in <city> that you can go outside that you don't run the risk of hitting a bump that's 2 inches….I bought it purposely for being in. You know, so I can pull up to a table and all that kind of thing. And you can't really have both. And I can't drive the scooter‐type. I have to have the joy stick. So this worked the best for me. I have another chair that someone can push to take me out. And so my biggest challenge is getting somebody to do that*. (CMB044)

In addition to travel and physical problems, some patients described challenges in organizing services. For example, a patient recounted how he had to press his family doctor to transfer his patient chart to a new doctor. Also, a patient who needed psychotherapy was given a list of 20 therapists by her family doctor, which she personally had to vet, to find a therapist. As demonstrated by this theme, there are small steps that patients have to take to access services. Although taking public transportation or transferring a file may seem routine or mundane by some, they are essential factors that are easy to overlook when considering how patients access care.

#### 3.3.2 Financial strain

Patients talked about feeling financially stressed because they needed to pay out of pocket for services or for incidental costs (eg, paying for parking at appointments) in order to access services. A patient described the consequences of out‐of‐pocket payment:

*You know, like even when I contacted ODSP [Ontario Disability Support Program] about paying the one‐time fee for the physio, they said no, we're not going to do it. So that's one of the reasons why I actually was on a waiting list, because they said you're on ODSP, you have to wait a year and a half. And within that year and a half, my bones didn't heal properly and I ended up back here [in the hospital] for a second time*. *(CMB 016)*

In summary, challenges at the patient level reveal how accessing care for some patients may mean taking on financial strain and stress. Also, the capacity to even arrange for and travel to services can be a problem for some patients. Health services, in some cases, were available to patients but this did not translate to services being accessible for patients.

### 3.4 Responding to the barriers to care

Some of the patients who were disappointed with the system and/or felt necessary care was not readily available, took matters in their own hands and responded by: “managing the system,” “making personal sacrifices,” “paying out‐of‐pocket for care,” “substituting with informal care,” or “resigning to system constraints”.

#### 3.4.1 Managing the system

Some of the patients who felt their care experiences fell short of expectations took explicit action by advocating for better care. One patient described how her daughter had persuaded a doctor to take her on as a patient:

*So I was very lucky that my daughter found the doctor she did. I was delayed a month coming because she couldn't find any doctor that would add me to his practice. And she went back to Dr. [family physician] and begged him*. *(CMB 044)*

Similarly, other patients had to “fight”, “persuade” and “persevere” to gain access to home care services, specialists and rehabilitation programmes. This theme is named “managing the system” because patients and their families felt they had to perform work‐arounds and make extraordinary efforts by negotiating the rules and regulations of the health system to access care.

#### 3.4.2 Making personal sacrifices

Some patients, who could not receive the services they required, had to travel long distances, or move closer to their formal care providers. For example, a patient who had temporary paralysis from the neck down explained why remaining at her local community rehabilitation hospital would have been her preference:

*Patient* *”Yes. I think it would have got me to where I am now if they had the funding.”*

*Interviewer* *”In what way would it have been better for you to stay?”*

*Patient* *”Just because my friends would be closer, and my sister. And because I'm from the area.” (CMB 022)*

#### 3.4.3 Paying out‐of‐pocket for care

Some patients purchased services, some of which were not typically considered “health” services, to manage living independently in the community. A patient with Guillain Barre Syndrome recalled arranging for snow removal:

*I had these 2 big snow banks but there's a crosswalk at the time there. And the guy came to clear it out so I gave him $20 and he took the bobcat and cleared my driveway for me. So yeah, I needed social assistance there…This was health care. It would have killed me to move all that snow*. (LM004)

Other types of supports patients paid out of pocket for included home care nursing, PSW care and speciality transportation services.

#### 3.4.4 Substituting with informal care

Patients' family members and friends were important sources of informal care when the formal system could not meet their needs. For example, a patient described how her son participated in her care:

*So my son had to come every morning before he was going to work. He came to give me a needle for 6 months before I went to the hospital…* (CMB001)

#### 3.4.5 Resigning to system constraints

Some patients appeared to resign to system constraints by characterizing their challenges as insurmountable. A patient, who needed help with bathing but did not qualify for public home care, described what led her to resigning:

*Interviewer* *”Do you feel that you would be able to get a friend to come in to help you?”*

*Patient* *”No. I would not ask a friend to do that.”*

*Interviewer* *”So basically you would have to hire if you needed to.”*

*Patient* *”I guess so, yes.”*

*Interviewer* *”And you're not happy with that?”*

*Patient* *”Not at all.”*

*Interviewer* *”Because you feel that you're entitled to that.”*

*Patient* *”I do, I do. I don't use the system very often so I think it's fair to make that one request and get it.”*

*Interviewer* *”Are you going to keep on fighting for that?”*

*Patient* *”Probably not.”*

*Interviewer* *”Because you've looked into it enough already.”*

*Patient* *”Yes.” (EH 008)*

In summary, “managing the system,” “paying out‐of‐pocket for care,” “making personal sacrifices,” “substituting with informal care,” and “resigning to system constraints” represent the ways in which some patients responded to barriers to accessing care. These actions illustrate the agency required to navigate health services and suggest patients may form adversarial relationships with the health system. The process of “responding to barriers to care” was also one which was fraught with frustration with the system and drove patients to “fight” for care.

## 4 DISCUSSION

This study's findings suggest that there are gaps and barriers in health services provision for people with multimorbidity and as a result, they experience considerable challenges trying to navigate the health system to access the services they need. In Ontario, the context of this study, publicly funded programmes such as home care, ambulatory care services and long‐term care, each have different sets of admission requirements (eg, means testing tools) and fall outside the universal, publicly funded entitlements of the health‐care system which is limited to hospital care and services provided by physicians for medically necessary care. Eligibility criteria that place the burden on the patient to demonstrate a threshold level of incapacity (often assessed at one point in time) may mean that principles such as ensuring that the “right” patient gets the “right” care may not be upheld. For instance, patients who fall just outside of thresholds such as income level, functional ability, age groups, may automatically be excluded for eligibility. We argue it is important that assessments include a degree of discretion; thus, patients who need services are not left without care because they fail to demonstrate eligibility although they clearly require the services.

Over a lifetime of managing various health conditions, some patients may have to go through different forms of means testing multiple times (some may even have to repeat the same testing on a regular basis). While it is important to have an accurate picture of a patients' status, the degree to which some patients feel strained by these processes of “gaining access” to care could be minimized. Integrating and sharing assessments across organizations is one approach to reducing redundant patient assessments. Another approach, prevalent in integrated models of care, is to have one professional (often times called a care/case manager) to coordinate services and assist the patient and their family members in navigating the health system..^34^ It is also important to ensure that assessments capture all information about a patient's circumstances, insight that can be garnered by simply asking patients what kinds of supports they need.

Findings from our study suggest that it is not enough to have services in place unless it is practical and realistic for patients to access them. When arranging services such as pain management clinics and diabetes management classes, providers should consider whether these kinds of interventions are appropriate and accessible for their patients. Patients face practical issues in travelling to treatments, such as parking costs, taking public transit, and navigating city sidewalks. Patients may need “non‐medical” supports to carry out their daily tasks, like an escort to appointments, or snow removal so that they can leave the house safely. In short, community services may be available but whether they are accessible depends on the complex dynamics between the personal circumstances of the patient, and their physical and social environment that ultimately shapes whether interventions are acceptable for patients and their families.

We postulate that the challenges that patients faced in our study may have been mitigated if they had a designated provider, such as a case manager, who was responsible for the coordination of services and was accessible and accountable for the actual delivery of the services. In Canada, an innovative model that considers these dynamics is the Program of Research to Integrate Services for the Maintenance of Autonomy (PRIMSA). PRISMA is community‐based integrated care system that uses private, public and voluntary health and social services to care for seniors with high needs in select communities in the province of Québec..^35^, ^36^ A case manager acts as the point person for the patient and their family to communicate with, in conjunction with a multidisciplinary team.^35^ An “individualized care plan”, tailored to fit the patient's needs and goals, is developed by the case manager, the care team, and the patients and caregivers, and the case manager is responsible for the coordination and the on‐going management of services.^37^

In light of the individualized contexts of how each patient experiences accessing care, we argue that any improvement strategies must first recognize that for some people with multimorbidity navigating the health system is like “fitting a square peg into a round hole”. Normative assumptions about who may need community services, such as the elderly or people with severe disabilities may mean younger people or those with only temporary or less complex impairments are excluded. Community‐based agencies are largely organized around delivering care to target populations, such as seniors, people with acquired brain injuries, and people with developmental disabilities. While parsing out resources by demographic group or health condition is likely to remain the norm in health services, we propose that this “siloed” approach to care needs to be reexamined because people with multimorbidity may not be able to access services as they do not fit into the narrowly defined thresholds of existing programmes.

This study also suggests that some patients are actively engaged with health services delivery and when faced with barriers, they may advocate, negotiate, and essentially “work the system” to access care. For example, some of the patients in our study who were denied access to services did not accept the status quo; instead, they persevered or coped by “managing the system”. Patients described an adversarial relationship with care providers and the health system. In fact, we found that people had to “fight” to gain access to necessary services like primary care, home care and physiotherapy services. Patients had to go beyond their roles as recipients of health‐care services and become advocates for what some described as “entitlements”. On the other hand, we also found the counter‐theme to managing the system—resigning to system constraints, or in other words “giving up the fight”, which may limit some patients' access in comparison to those who actively seek workarounds. Both themes suggest patients who are ill, may not only have to manage their ailments but also manage the system. In consideration of Penchansky and Thomas'^15^ conceptualization of access as the “fit” between patients and services, our findings indicate patients who do not “fit” are either left without services or those who are able to access services have to alter their personal circumstances (eg, incurring financial strain) to ‘fit’ the system.

Patient—health‐care services interaction is the core of Levesque et al.^17^ framework for access to care and presents the process of obtaining care (perceiving, seeking, reaching, paying and engaging) as “abilities”. We agree that their conceptualization of access is analytically useful, as findings from our study reveal types of “work” which requires “abilities” to access care. Indeed, some of the themes in our study map onto Levesque et al.^17^ conceptual framework. For example, the theme of “financial stress” from this study, meets their definition of the “ability to pay”. Our study expands on Leveque and colleagues^17^ conceptualization of “ability to engage”, characterized as the ability to participate and be involved in decision‐making, because findings from our study suggests that patients may need to employ more covert ways of engagement, such as “working the system” to access care.

In addition to patients' abilities, it is important to consider the context which necessitates the deployment of such abilities. Especially salient to the dynamics of patient—health services interaction are the health policies and health system arrangements that create the conditions in which patients feel like they have to fight, struggle and negotiate to gain access to care.

## 5 LIMITATIONS

One of the methodological challenges inherent to a secondary qualitative analysis is that researchers need to address the extent of the difference between the primary study's focus and the secondary analysis' research question.^28^ The primary study was a large‐scale mixed methods study,^27^, ^38^ of which the issue of challenges to accessing care in the community comprised just one question in the interview guide. The interview guide for the primary study was fairly structured and broad. We attempted to capture as much as we could within the data set related to our research question. Although there were rich responses to the types of challenges patients experienced, the feelings and reactions of patients were not fully captured by the interviews. However, we were able to elicit some insight into patient responses as illustrated in the “responses to barriers to care” theme. Also, it is important to note that the primary study's interview guide's wording of the question: “have you experienced any challenges in accessing care in the past?” may have led interview participants to recount only negative experiences, rather than eliciting more balanced responses.

## 6 CONCLUSION

Patients with multimorbidity have to contend with a host of system and individual level barriers when endeavouring to access services in the community. The barriers to qualify for and actually receive services require patients to go beyond the role of health‐care recipients. Responding to systemic barriers and limitations in administration and service delivery, patients who are already vulnerable due to complex health conditions have to advocate for necessary services, make significant changes to their lives, pay for care or rely heavily on informal, unpaid sources of support (eg, family caregivers). The availability of non‐clinical services or social services, within and outside health systems, play an important role in determining whether patients are even able to access medical interventions; thus, patient assessments must consider these factors. Shifting the lens to improving patient experience is an essential consideration in improving chronic disease management. Systemic barriers that have serious implications on patients' lives need to be addressed in ways that considers patients' individual circumstances and respects their roles as partners and does not treat them as adversaries.
